# Spelling Profile Assessment (SPA): A new spelling test for English primary school children; standardization

**DOI:** 10.3758/s13428-026-03195-7

**Published:** 2026-11-09

**Authors:** Georgia Niolaki, Harry Purser, Aris Terzopoulos, Janet Vousden, Saskia Kohnen, Jackie Masterson

**Affiliations:** 1 University of Birmingham, Birmingham, UK; 2 Birmingham City University, Birmingham, UK; 3 Nottingham Trent University, Nottingham, UK; 4 Australian Catholic University, Australian Centre for the Advancement of Literacy, Sydney, Australia; 5 Institute of Education, UCL, London, UK; 6 School of Education, University of Birmingham, Edgbaston, Birmingham, 0044 7881594 772 UK

**Keywords:** Primary spelling ability, Phonological knowledge, Morphological knowledge, Orthographic knowledge, Psycholinguistics, Psychometrics, Spelling assessment

## Abstract

We present the construction, psychometric evaluation, and administration of a new single-word spelling battery that includes: a. irregular (e.g., yacht) and regular (e.g., trap) words and pseudowords (e.g., knep) (*N* = 87), and b. a morphological spelling test with real words (20 affixes/seven suffixation rules) and pseudowords (mirroring the rules of the real words) (*N* = 40). After the pilot phase, the final battery was standardized on 1053 children (Year_R to Year_6 F = 514). The battery across tasks presents outstanding reliability (.97 to .99), and criterion and convergent validity were compared with the Wechsler Individual Achievement Test-Third UK Edition (Wechsler, 2009) spelling subtest. The Spelling Profile Assessment (SPA) battery provides standard scores (mean = 100, *Sd* = 15) per subtask, a total composite standard score for each test (a and b) and 95% confidence intervals. We calculated age-based standard scores using a continuous developmental trajectory rather than assigning individuals to fixed age groups. We model gradual developmental change to provide a more precise score. The test also includes a stopping rule, which streamlines its administration and reduces stress for children who can only spell a limited number of items. Additionally, we automated every step of the standardization process and made it available under open access, ensuring transparency, reproducibility, and ease of adaptation. This tool contributes to the spelling literature by offering a comprehensive battery for assessing phonological, orthographic, and morphological spelling skills in primary-age children. It can serve as a valuable tool for educators/clinicians to gauge students’ progression and tailor their spelling instruction accordingly.

**Supplementary Information:**

The online version contains supplementary material available at https://doi.org/10.3758/s13428-026-03195-7.

## Introduction

Conquering English spelling is not an easy task, and some may not be able to master this linguistic skill even in adulthood (Allyn & Burt, 1998). Weaknesses in spelling can also negatively impact written expression and academic self-concept (Graham & Santangelo, 2014; Puranik & Al Otaiba, 2012). This is particularly pertinent for those with diagnosed reading and spelling difficulties such as dyslexia, with studies providing evidence of co-occurrence with mental health conditions such as depression and anxiety (Xiao et al., 2023; Niolaki et al., 2025). Job applicants with poor spelling are more likely to be rejected by recruiters (Martin-Lacroux, 2017) and are potentially viewed as having lower levels of conscientiousness, interpersonal skills, and cognitive abilities (Sterkens et al., 2023). Hooper et al. (1994) stated that “*impaired spelling and writing has the potential to result in daily embarrassment or humiliation at school*” (p. 401).

Difficulties with achieving spelling targets are often evident early in school-aged children. For example, more than a quarter of 5-year-olds in England – 187,000 children – did not meet the expected standard for literacy, including spelling, in 2022/23 (Rodriguez Cabrera et al., 2024), p. 4). In addition, in the UK, only 72% of pupils met the expected grammar, punctuation and spelling standards, unchanged from 2023 (GOV.UK, 2024). This is despite findings by Ofsted (Office for Standards in Education, Children’s Services and Skills) that most schools in England explicitly teach spelling and letter formation, mainly through the phonics curriculum (GOV.UK, 2024, p. 12–13). Similar low trends in spelling ability are reported elsewhere, with national and international testing data indicating that many students do not meet expected standards (e.g., the Australian Curriculum, Assessment and Reporting Authority (ACARA), 2024). Teachers often say they lack explicit guidance on how to support spelling because the curriculum is vague and schools do not always have a spelling policy (Esposito et al., 2023). Many of the UK government-approved Systematic Synthetic Phonics (SSPs) programs have a spelling component, but they are not rigorously tested or supported by quality evidence.

Evaluating whether spelling is taught successfully requires ongoing monitoring of student achievement. To this end, we aimed to create a rigorous, theoretically driven spelling battery that supports nuanced assessment of different spelling skills from the beginning of school entry. Our test (SPA) assesses the spelling of regular, irregular, and non-words (part a), as well as the spelling of frequent morphological generalizations (part b) as a Tier 2 and 3 measuring tool.

### The English writing system

*‘English spelling teases the brain and twists the tongue’* (The Spelling Society, 1999, p.1). This phrase beautifully captures the challenges and quirks of the English orthography that make it both mentally stimulating and physically tricky to spell. George Bernard Shaw explained cleverly that ‘fish’ can be easily spelled “ghoti” as ‘gh’ is pronounced an ‘f’ in ‘tou*gh*’, ‘o’ as an ‘i’ in ‘w**o**men’, and ‘ti’ as a ‘sh’ in na**ti**on (The Spelling Society, 1999). However, this example is often criticized for ignoring position, word origin and other complexities that govern the English orthography.

The English orthography is well known for its lack of regularity, meaning that spelling of sounds is not always predictable and alternative representations may exist (such as *two, to, too, or there, their, they’re*). Although most English words can be at least partially spelled correctly using phoneme-grapheme correspondences, many contain one or more sounds that are not spelled with their most common letters. Ziegler et al.’s (1997) analysis showed that 72.3% of English monosyllabic words contain some degree of spelling irregularity. Many of the most common words in English are part of this category (e.g., *was, who, would,* etc.) (Stuart et al., 2003). Stuart et al. (p. 549) define irregularity as *words that do not adhere to the phoneme-grapheme correspondence rules in English*.

Successful spelling requires the integration of many different skills outlined next (e.g., Kohnen et al., 2015; Niolaki et al., 2020; 2022). These include phonology (sublexical skills), orthography (lexical skills), morphology and semantics. Successfully encoding new or unfamiliar words (e.g., new science terms like ‘zygomatic’, which pupils in secondary school must regularly learn) requires good sublexical phonological skills. While adequate lexical skills facilitate the spelling of known words, they are the only way to spell less predictable words such as ‘*debt*’ or ‘*gauge*’. Semantic knowledge is required to choose the correct homophone when spelling words such as meat/meet, knight/night or two/too/to. Knowledge of morphological spellings allows for successful spelling of the many morphologically complex words in English (e.g., unsatisfactory) (for a detailed reference, see Colenbrander et al., 2020). Given the significance of these forms of knowledge, teaching these skills (phonology, orthography, morphology and semantics) and consistently tracking their acquisition throughout the academic year is crucial.

### The assessment of spelling *a means to an end?*

For educators and researchers to effectively support spelling development, it is essential to have a tool that is both theoretically grounded and sensitive enough to capture distinct spelling competencies, including phonology, orthography, morphology, and semantics. This is because accurate spelling is not a uni-dimensional skill. It is a complex skill that requires multiple abilities to work in tandem. Ideally, tests should be based on theories related to the skill they are assessing. This is important as it ensures that the test actually measures what it intends to measure. Theory-based assessments are more likely to evaluate the cognitive processes that are involved in the relevant skill, in this case, spelling. As such, we will briefly review some of the relevant theories on spelling.

One commonly mentioned theory in spelling is the triple word form theory. Triple word form theory purports that multiple language skills interrelate to produce the final spelling outcome (Garcia et al., 2010; Bahr et al., 2012). Empirical evidence from children’s spelling production tasks suggests that learners make errors reflecting the three linguistic areas of phonology, orthography, and morphology (Bahr et al., 2012). Specifically, they suggested that learners draw on the analysis and retrieval of phonological, orthographic, and morphological elements from orthographic memory. Research on children with dyslexia and typically developing spellers suggests that all three linguistic areas are relevant to assessing and remediating literacy difficulties (Berninger et al., 2001; Berninger & Fayol, 2008). Thus, we consider that our spelling test should be developed to align with these linguistic elements that underpin spelling skills. Indeed, these elements are considered in most current theories of spelling and spelling acquisition. However, although semantics is implied in the triple word theory as part of morphology and orthography, its status is not clear in the theory. In addition, Garcia et al. (p. 89) noted *that the theory does not specify how children of differing spelling ability may orchestrate or coordinate the different kinds of linguistic awareness so that links among orthographic, phonological, and morphological representations are created in the internal lexicon, as initially described by Amalgamation Theory* (Ehri, 1978), meaning that we need a more precise framework that explicitly outlines how spelling is processed from seeing the word (e.g., when copying) or hearing (when spelling to dictation) to spelling it.

The integration of multiple patterns theory, put forward by Treiman and Kessler (2014) might offer some insights here. The theory is based on the idea that linguistic representations of spelling contain a variety of patterns: context-free phoneme-grapheme correspondences (PGC), context-sensitive patterns, graphotactic and morphological patterns. Using the word ‘pattern’ acknowledges the fact that English rarely has reliable all-or-nothing rules, but rather common occurrences of certain spellings in particular contexts (e.g., /e/is often – but not always – spelled as <ea> before <d>). Some of these patterns may be taught explicitly (e.g., context-sensitive spellings like the <ead> in <head>), while others may be acquired through exposure, that is, implicitly. The theory predicts that for words where multiple patterns all point in the same direction, such as in the spelling ‘dog’, spelling will be easier. In contrast, words are harder when patterns diverge, like ‘yacht’ (i.e., context-free PGCs would support the spelling <yot>). The theory has largely been validated using corpus studies by identifying spelling patterns in English (e.g., Altmiller et al., 2023; Treiman & Kessler, 2023). Additional work has been conducted to examine spellers’ sensitivities to the patterns detected in the orthography (e.g., Cassar & Treiman, 1997; Treiman & Boland, 2017). This theory, in particular its corpus analyses, makes meaningful contributions to our understanding of the patterns that children need to learn. However, to the best of our knowledge, there are currently no studies that investigate the predictions of this theory in low-skilled spellers.

**Fig. 1:**
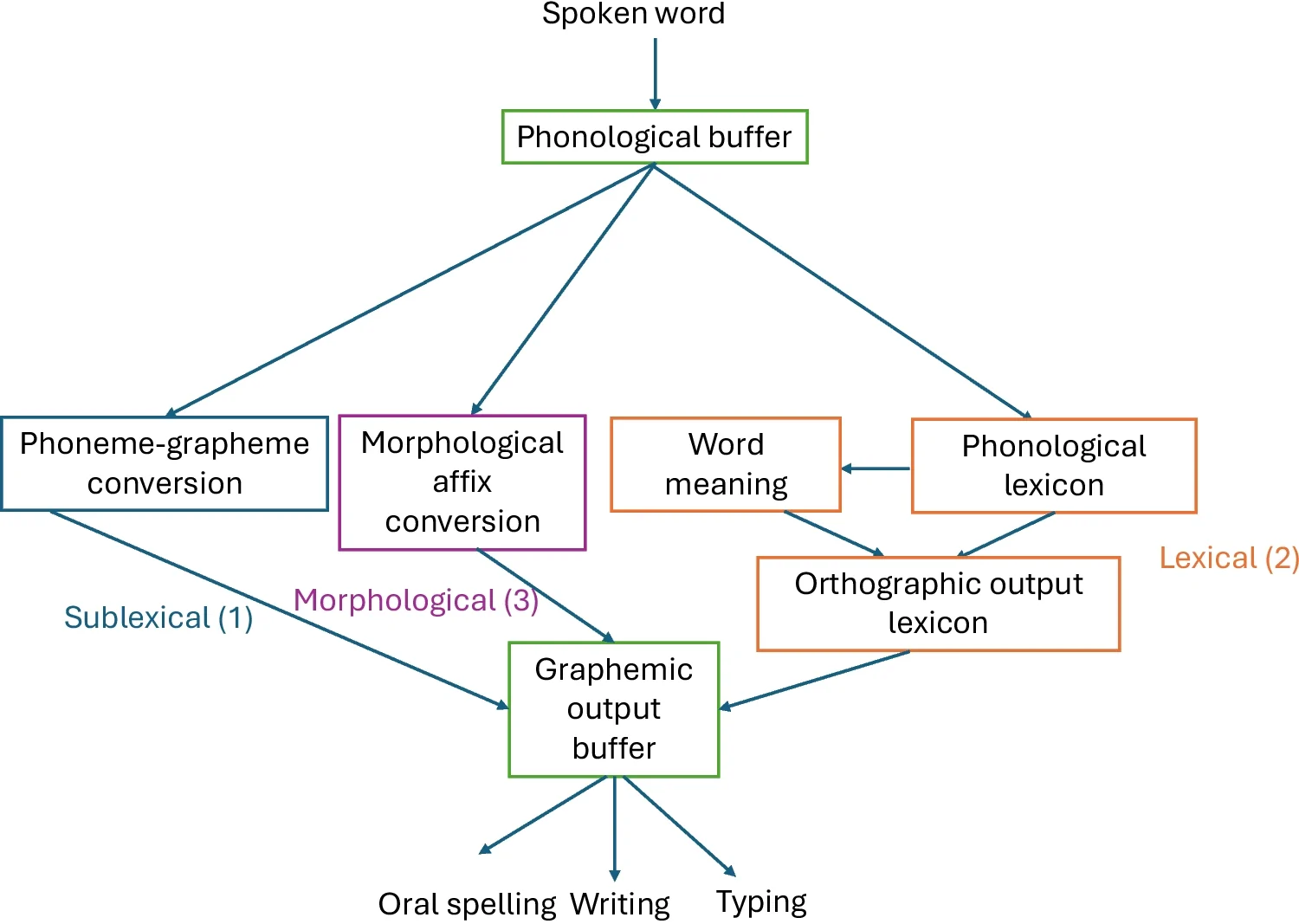
The dual-route model of spelling adapted to include morphology

Based on their success in formulating hypotheses about impaired spelling in pediatric populations (e.g., Coltheart & Kohnen, 2012), the current test was largely based on dual-route models of spelling (Barry, 1994; Tainturier & Rapp, 2001). When first published, the main idea of these models was that spelling can be accomplished via two broad pathways: One relies upon computing spellings based on mappings between phonemes and graphemes (the ‘sublexical’ pathway (1) in Fig. 1) and the other references long-term orthographic memories (the ‘lexical’ pathway (3) in Fig. 1). In addition to long-term memories for spellings of words, the lexical pathway also includes access to word meanings (semantics). Sublexical spelling accomplishes accurate spellings for words with predictable phoneme-grapheme mappings (e.g., *dog*, *wombat*). It can also compute phonologically plausible spellings for unknown words, including nonwords (e.g., *nop*, *lumgad*) and words not yet in the orthographic lexicon (e.g., new vocabulary, spellings of words children know only in their spoken form). All known words can be spelled accurately via the lexical pathway, but the lexical pathway cannot produce spellings for unknown words, including nonwords. In contrast, the sublexical path cannot compute accurate spellings for words that include unpredictable phoneme-grapheme mappings. In addition, semantic access along the lexical path is required, for example, to produce the correct spelling for a homophone (e.g., *two, too, to*).

Dual-route models are better known in the domain of reading, where recent research has produced extensions to include processing of morphologically complex words (e.g., Güven & Friedmann, 2021). While, to our knowledge, not generally depicted in published work on dual-route models of spelling at this point (but see Haluts & Friedmann, in press, for relevant experimental work), extending dual-route models of spelling to include morphology is important (e.g., Treiman, 2017). There are several ways in which morphology may be represented in models of spelling. It is possible that morphologically complex word forms are part of the orthographic lexicon (Verhoeven & Perfetti, 2011). Alternatively, morphology might form its own pathway, similar to what has been suggested in models of reading (e.g., Güven & Friedmann, 2021). A separate pathway (see *morphological pathway* (3) in Fig. 1), would fit well with the observation that spellers are able to correctly apply morphological patterns when spelling unknown words (e.g., “Yesterday, she/trokt/,” with the nonword spelled as <trocked>), as evident in the morphology part of the current test and other work (e.g., Breadmore & Carroll, 2016). When writing words to dictation, spellers may decompose spoken words into affixes and bases and convert these spoken forms into their written forms^1^. While known bases are likely stored in the lexicon (e.g., Breadmore & Carroll, 2016), affixes are processed via the affix conversion route. Targeted experimental work, including in the form of possible dissociations between skills (e.g., nonword spelling intact with impaired morphological spelling skills) in impaired populations to validate morphological extensions of dual route models of spelling, has not yet been published to the best of our knowledge.

Mapping directly onto dual route models while taking into account examinations of corpus data (Treiman, 2017), the SPA (parts a and b) includes four subscales:

1. Nonwords were included to assess children’s ability to apply phoneme-grapheme correspondences. As such, nonwords were included in the test to evaluate children’s acquisition of the sublexical spelling route. This is part of what forms phonological spelling knowledge. For example, accurate spelling of the nonword <chust> requires that children segment the full spoken form into its phonemes, which are then mapped to graphemes.
2. Irregular words were included to assess children’s ability to form word-specific orthographic representations in the lexical route. This is part of what forms orthographic spelling knowledge. For example, the word <people> the spelling of /i/ in the first syllable is not predictable by common, context-free, nor context-sensitive phoneme-grapheme correspondences. As such, accurate spelling relies upon long-term orthographic knowledge in the orthographic lexicon along the lexical pathway.
3. Morphologically complex items were included to assess children’s ability to apply morphological spelling patterns. This is part of what forms morphological spelling knowledge and may be represented in the orthographic lexicon for words and along an additional morphologically focused spelling route (which has to be used when spelling morphologically complex nonwords). For example, to spell the past tense in the target nonword <trocked> in the sentence “Yesterday she /trokt/,” correctly, children need to process the fact that the target is a past tense verb, and that the past-tense morpheme is spelled ‘ed’. This cannot occur via the lexical pathway, as children will not have heard this nonword before.
4. Regular words assess children’s ability to apply phoneme-grapheme correspondences, apply graphotactic patterns, and word-specific orthography. As such, it assesses a different aspect of the sublexical route from nonwords. For example, ‘shelf’ represents an item where accurate spelling requires a single L and a single F. This is in contrast to spellings such as <shell> and <stuff> where the L and F are doubled. Graphotactic rules/patterns or context-specific spelling knowledge make these spellings predictable. We propose that this knowledge is represented along the sublexical pathway. However, all known words can also be spelled correctly via the lexical pathway. A regular word such as ‘blade’ would not be classified as an irregular word; however, only one translation of /ei/ produces the accurate word spelling: <a-e>, the spelling <ai> (as in *laid, raid*) is not correct for the word *blade*. Hence, accurate spelling could either occur via selection (possibly based on frequency) of the correct spelling <a-e> along the sublexical pathway (with other spellings also being represented here; e.g., <ai>), or via lexical knowledge.

Taken together, the five spelling subscales of the SPA allow an overview of the most relevant aspects of spelling knowledge that make up the English orthographic system and are represented in current theories of spelling.

### Why is this spelling battery important compared to others?

Our spelling tool fulfills an important need in teaching spelling, as it can be used as an early spelling assessment, even for Reception (R) Year learners, which is not the case for many other spelling tools that identify needs for older learners (8 years old and onwards). While targeted intervention can produce changes even in the later years (e.g., university students Ocal & Ehri, 2017), early identification facilitates early intervention while the brain is more receptive to change (Nelson et al., 2024; Niolaki et al., 2020). Detecting difficulties early may also mitigate the potentially detrimental impact on the learner’s academic and mental well-being. The SPA battery includes real words (*SPA single_word test*) (regular and irregular words concerning sound-letter associations) and pseudowords, which were created by transposing regular words so that we have items that follow the English spelling rules (orthographically valid items). Regularity can be conceptualized as a continuous measure (e.g., Colenbrander et al. 2020), partly depending on what context or rules are assumed when encoding sound to print. At one extreme, some words contain transparent one-to-one correspondences, such as mat, which can be spelled by mapping phonemes, context-free, onto their most frequent graphemic representations. Other words, such as *fuss*, are semi-transparent because they require context-specific knowledge (the ‘floss’ rule) to be spelled correctly. There are also less-transparent or opaque words, like yacht, where reliance on phoneme-grapheme correspondences or common rules cannot lead to accurate word spellings. While we acknowledge that this continuum exists, the SPA regular words are those that can be spelled by mapping phonemes onto their most frequent graphemic representations, relying upon some context-sensitive mappings (e.g., /k/ as <ck> in check), while the irregular words are those that cannot be spelled by mapping phonemes, context-free or context-sensitive, onto their most frequent graphemic representations. The morphological spelling test (*SPA Morph_test*) includes three out of the four phases proposed for teaching morphological rules by Colenbrander and Kohnen (2021, personal communication) (we expand on this in the *Measures* section).

SPA provides information about these different spelling dimensions all in the same test battery. As a consequence, teachers and researchers can conduct all testing with a single tool rather than requiring multiple tools to assess different spelling processes and identify which spelling area (phonology, orthography, morphology, or semantics) needs tailored instruction (Kohnen et al., 2009). This matters because it is not unusual for children to struggle in only one of these areas of spelling (Kohnen et al., 2015). Also, we use pseudowords (made-up words that sound like real words) in both parts of the test (*SPA single-word* (a) and *SPA Morph_test* (b)) as pseudowords purely tap non-lexicalized processes, and they can ensure measurement of generalizability to untaught/unfamiliar items. Pseudowords can reveal the child’s knowledge of phonology and spelling patterns without the influence of meaning and prior word-specific knowledge (Kohnen et al., 2009).

In our spelling test, we avoided compiling the items on the basis of difficulty or predictive value. We also avoided old practices where educators sought to identify children who struggled with spelling by using so-called ‘*demon lists’* of words which are neither theoretically motivated nor reflecting usage (Cahen et al., 1971). SPA items were selected first to be representative of one of the spelling skills described above. They also featured in the UK National Spelling Curriculum; in that way, we ensured that there is pedagogical value. The items are balanced regarding frequency, length, and other item-related characteristics such as contextual diversity (how many times the item appears in children’s books) and phonological neighbors (words that are similar in phonological structure, for example, phonological neighbors of best would be ‘nest, bust, bent, test’…). We also evaluated the items for concreteness, meaning that they can easily or not evoke sensory experiences, and imageability, the words’ capacity to evoke visual images (experiences) (Brysbaert et al., 2014). Both skills are considered to be associated with memory and with reading (Paivio, 2013; Payne et al., 2015). A carefully curated list, based on word frequency and items taught in the UK national curriculum, is a more effective tool for identifying weak spellers and predicting which spelling skill (e.g., sublexical, lexical, morphological, see Fig. 1) is likely to pose greater difficulty (Niolaki et al., 2022). In addition, balancing across psycholinguistic variables is valuable, as we know these can influence performance across the different word lists (i.e., controlling for difficulty across the lists).

In addition to careful preselection, we conducted a tailored pilot study of the SPA single-word test, collecting data from 641 children in Reception through year 6. Item response theory (IRT) enabled us to evaluate item characteristics and refine a more concise test version (for details, see Niolaki et al., 2019). We did not apply the same procedure (pilot phase) to the SPA Morph_test, as our prior experience with the SPA single-word test informed our approach to selecting items for this additional part of the test. From the outset, we opted for a shorter version consisting of 20 items; reducing the number further would compromise the test’s ability to capture the full complexity of morphological spelling patterns.

While, without a doubt, other great spelling tests exist, the SPA adds unique value and fills a current gap in spelling assessments. Several recent tests, like the Test of Orthographic Competence (TOC, Mather et al., 2024), are excellent tests that evaluate mainly orthographic competence using a mixture of regular and irregular items. Also, the recent Test of Dyslexia (ToD, Mather et al., 2008) assesses regular and irregular single-word spelling in separate lists, but not pseudoword spelling. The latter test has a good age range, but the former is from age 8 onwards, and both tests have been standardized in the USA. Also, written expression tests are not a good measure of spelling skills, as the learner will avoid items that they struggle to spell and will use less sophisticated and challenging vocabulary. Howell et al. (1993) recommend that a learner needs to make more than 75 errors in a free writing task for meaningful spelling error analyses to occur.

In summary, the SPA single-word and SPA Morph_test are theoretically motivated tests with carefully selected and statistically motivated items which allow early identification of different types of spelling difficulties, all within the same test.

### Purpose of the present study

A consistent observation in educational assessment is that children’s performance on literacy and language tasks varies in mean level and variability across development (Rittle-Johnson & Siegler, 1999; Treiman, 2017; Varnhagen et al., 1997). We opted for a methodology that will produce age-based standard scores incorporating a continuous developmental trajectory, rather than relying on discrete binning alone (Carlin & Louis, 2008). This approach was used to minimize abrupt cut-offs between age groups and to capture natural increases in score variability as children develop their spelling skills. By enforcing a smooth progression of means and variances with age, the resulting norms allow for more precise comparisons of individual performance, especially in cases where sample sizes in specific age brackets are modest (Gelman & Hill, 2007). Importantly, every step in the standardization analyses, from raw data processing to the creation of the final lookup sheet, was fully scripted using openly available code (that can be accessed here). This end-to-end automation can ensure complete transparency, reproducibility, and ease of adaptation.

Thus, the present study portrays the development and standardization of the SPA battery, SPA single-word, with the added element of the Morph-test. We present the tests’ characteristics, the new scale’s validity and reliability, and the tool’s standardization. In the Appendix, we include the pilot phase outcome. The supplementary tables, which report the standard scores per word list, the standard error of measurement (SEM) with a 95% confidence interval (CI), and the composite standard scores, can be accessed in the open science document (https://osf.io/vfp7h/overview).

## Methods

### Participants

#### Part (a) SPA single-word

Participant recruitment aimed to achieve a representation of children with different socio-economic backgrounds and ethnic and gender diversities and needs. Children came from multiple schools (Rural/Urban state, Church of England (CoF), private) to ensure a broad mix of backgrounds and proficiency levels (22 different schools in total). Each school had between 150 and 500 students, and children came from 65 different classrooms. Schools were chosen from large cities such as Birmingham and London and smaller regions/towns/villages.

All participants were between 5 and 12 years old, thus spanning a critical developmental window for language and literacy skills across all of primary school (Nelson et al., 2024). Each child’s age in months was recorded, and individuals older than 144 months or younger than 60 months (5 years) were excluded to preserve a consistent age range (one participant was excluded). Educators at each site provided class lists, from which participants were selected after their parents/caregivers and themselves consented to participate in the study, provided they had no reported uncorrected hearing or visual impairments or spelling anxiety (according to the Special Educational Needs Coordinator (SENCo), feedback). A final total of 1016 participants remained after exclusions Table 1.

**Table 1:** Demographics of the total sample

Yr R	Yr 1	Yr 2	Yr 3	Yr 4	Yr 5	Yr 6
138 F = 41% WE = 73% EAL^1^ = 24% SEN = 9%	55 F = 44% WE = 44% EAL = 19% SEN = 14%	127 F = 50% WE = 50% EAL = 50% SEN = 4%	235 F = 50% WE = 74% EAL = 22% SEN = 6%	153 F = 58% WE = 67% EAL = 28% SEN = 4%	164 F = 48% WE = 73% EAL = 25% SEN = 6%	144 F = 46% WE = 36% EAL = 52% SEN = 10%
UK general school_data (Department for Education in the UK (DfE), 2024a, b)						
WE = 65.5%	EAL = 39%	SEN = 5%				

Yr = Year in the UK (Grade), F = female in a binary categorization, WE = white English, EAL = English as an Additional Language, SEN = Special Educational Needs, ^1^ = These are based on the whole sample before trimming, because the digital records for EAL are incomplete, and we did not use a specific measure for EAL

### Part (b) SPA Morph_test

The additional SPA Morph_test was standardized using a smaller number of the same children who participated in standardizing the SPA single-word test. This sub-sample included 399 students from the SPA single-word sample. These students came from seven different schools and attended 34 different classrooms. Other than non-white and EAL participants, we have good representation for the SPA Morph_test (see Table 2). This difference is not unexpected, as we have a smaller sample size and fewer schools (seven schools in total, whereas the total sample had 22 different schools).

**Table 2:** Demographics of the SPA Morph_test sub-sample

Yr R	Yr 1	Yr 2	Yr 3	Yr 4	Yr 5	Yr 6
38 F = 29% WE = 97% EAL = 9% SEN = 13%	35 F = 43% WE = 100% EAL = 18% SEN = 23%	64 F = 47% WE = 98% EAL = 7% SEN = 8%	73 F = 41% WE = 86% EAL = 12% SEN = 19%	67 F = 66% WE = 85% EAL = 25% SEN = 9%	72 F = 46% WE = 92% EAL = 6% SEN = 12%	50 F = 48% WE = 90% EAL = 11% SEN = 12%

Yr = Year in the UK (Grade), F = female, WE = white English, EAL = English Additional Language, SEN = Special Educational Needs

### Measures

#### The initial SPA single-word test list

The initial SPA single-word test included 36 irregular items, 36 regular items, and 34 pseudowords (the list was published by Niolaki et al., 2022). Research assistants selected initial items from the UK National Spelling Curriculum (DfE, 2013). A second phase (clearing) of item selection was conducted after the first and fourth authors reached an agreement on the suitability of the chosen items. Additionally, each item’s psycholinguistic characteristics were analyzed. We used two databases for frequency and contextual diversity values (Masterson et al., 2010; Van Heuven et al., 2014). For phonological neighbors, we used N-Watch (Davis, 2005). We also calculated the length of each item’s phonemes, letters, and syllables. In total, we had an equal number of items that were short (e.g., *said, list*), medium (e.g., *grand, science*), and long (e.g., *yesterday, station*), and for each length category, we selected an equal number of items with low, medium, and high frequency (see Appendix D). A Kruskal–Wallis test was conducted to compare psycholinguistic variables (frequency, length, contextual diversity, and phonological neighbors) across the three word-types (Appendix D). The results indicated no significant difference between groups, *p* >.08 across variables and groups.

#### Pilot of the initial SPA single-word test

We conducted a meticulously designed pilot study exclusively for the SPA single-word test, gathering data from 641 children (these are different children from the SPA battery sample) across Reception to year 6 in the UK from six primary schools between 2016 and 2019. Utilizing item response theory (IRT), this analysis enabled us to assess item characteristics and create a more concise test version (as detailed in Niolaki et al., 2019). The two-parameter logistic (2PL) model vs. 1PL resulted in excellent fit;* χ*^*2*^*irregular* (df35) *= 288, p* <.0001; *χ*^*2*^*regular* (df35) *= 624, p* <.0001 *χ*^*2*^*pseudowords* (df33) *= 181, p* <.000. The item characteristic curves (ICC) per type of word are provided in Appendix A. In addition, the initial pilot item selection (total SPA single-word spelling list) showed excellent reliability (mean = α >.94) and criterion and convergent validity, as each test category was correlated with the WIAT 2 (Wechler, 2009) single-word spelling test (irregular, regular, and pseudoword items) (*r* >.57, lowest between pseudowords and WIAT 2 single-word spelling) (see Appendix B and C). Interestingly, irregular words and pseudowords received the lowest level of association, *r =.53;* this is not unexpected according to the theoretical framework (dual-route model) as these two lists tap different processes (lexical/semantic and sublexical). Further, if there were a perfect association, the need for new test items might be questionable.

#### Final SPA single-word test development

The new spelling test, after the pilot phase, had 29 irregular, 29 regular, and 29 pseudoword items. These items were used to standardize the tool. Next, we present the characteristics of the SPA single-word instrument. Similar to the pilot phase, we had an equal number of short, medium, and long items that matched in frequency (low, medium, and high). We carried out the Kruskal–Wallis test, which indicated that the final item sets were matched across the different word types (irregular and regular) in length, frequency, contextual diversity and phonological neighbors, concreteness and imageability (see Table 3). The last two psycholinguistic variables are drawn from an adult database, as such measures do not exist in children’s written vocabulary datasets. Overall, regular and irregular words had almost the same number of items considered abstract and rated zero (10 and 9, respectively).

**Table 3:** Psycholinguistic characteristic per item type (irregular, regular and pseudowords)

	Log_ CPWD_a_	CBeebies_b_	CBBC_b_	Zipfreq_b_	N Letters	N Phonemes	N Syllables	CD zip_b_	Phon N_c_	Imageability_c_	Concreteness_d_
Irregular items	2.02(1.3)	4.3 (1.4)	4.6 (1.3)	4.7 (1.3)	6.5 (1.8)	4.9 (1.6)	2.0 (.86)	.30(.34)	5.5 (5.7)	441 (135)	276 (228)
Regular items	1.5(.77)	4.1 (.94)	4.2 (.96)	4.3 (.95)	6.2 (2.0)	5.43 (1.77)	1.7 (.79)	.18(.22)	6.3 (6.8)	443 (103)	258 (252)
pseudoword	-	-	-	-	6.5 (2.0)	5.6 (1.9)	1.8 (.8)	-	-	-	-
Kruskal–Wallis *H*	.66	.58	3.1	1.8	55	1.5	3.2	2.2	.01	.26	.12
*p*	.41	.44	.08	.17	.75	.47	.19	.13	.9	.87	.73

a = Masterson et al. (2010); b = Van Heuven et al. (2014); c = Davis (2005); d = Coltheart (1981)

When the initial methods and results were presented at the Forum for Research in Language and Literacy conference in the UK (June 2019), colleagues noted that the irregular word list might be particularly challenging for Reception Year children. They also expressed concerns that this difficulty could impact children’s confidence in their spelling abilities. As a result, a decision was made to include a few high-frequency words that most children would have mastered by the end of the Reception Year, such as *said, was, have,* and *come*. The remaining word lists and items only received positive comments, including on the need for such a test that can be effectively used in other English-speaking countries (such as the USA, Canada, and Australia, but also when English is taught as a second language).

Apart from the balanced psycholinguistic traits, the fact that the items came from words taught throughout Primary school and were expected to be mastered by learners at the end of Primary, the real words were also balanced in morphological complexity. In the irregular word list (with items that, as a whole or in some part, do not use the most probable letter/s in the written language, e.g., *said*), we had 18 morphologically simple words and twelve complex ones. Similarly, the regular word list included almost the same number of morphologically simple and complex words. This adds value when assessing a student’s understanding of spelling rules beyond just phonological and sublexical orthographic elements. See Table 4 for a representation of the morphological complexity and simplicity of the items.

**Table 4:** Morphologically simple and complex words in the SPA single-word list (irregular and regular items)

Irregular		Regular	
Monomorphemic	Morphologically complex words	Monomorphemic	Morphologically complex words
**• was** **• said** **• have** **• come** **• people** **• other** **• please** **• dairy** **• young** **• language** **• wheat** **• shoulder** **• autism** **• country** **• science** **• search** **• nature** **• aspire**	• deadly → *dead* (base) + *-ly* (suffix) • remember → *re-* (prefix) + *member* (base) • station → *stat-* (root from Latin *stare* meaning “to stand”) + *-ion* (suffix) • fortune → *fort-* (root meaning "luck") + *-une* (suffix) • flavor → *flav-* (root from Latin *flavus* meaning “yellow”) + *-our* (suffix) • neighbor → *neigh-* (near) + *-bour* (dweller) • marvelous → *marvel* (root, meaning "wonder") + *-ous* (suffix, forming an adjective) • absolutely → *absolute* (base) + *-ly* (suffix, forming an adverb) • societies → *society* (base) + *-es* (plural suffix) • analyze → *ana-* (prefix, meaning “up” or “back”) + *-lyse* (root, meaning “to loosen or break down”) • councilor → *council* (base) + *-or* (suffix, indicating a person)	• cup • best • clip • stem • fond • life • strong • grand • scram • shelf • check • prize • scarper • trombone • lantern • ground • blade	• swept → the word “swept” is a complex morphological word because it is the past tense and past participle of the verb “sweep.” • without → *with* (base) + *out* (base) • sporty → *sport* (base) + *-y* (suffix, forming an adjective) • understand → *under* (prefix) + *stand* (base) • subside → *sub-* (prefix, meaning "under") + *side* (base) • yesterday → *yester-* (prefix, meaning “previous day”) + *day* (base) • impact → *im-* (prefix, meaning “into”) + *pact* (root, meaning “agreement or fasten”) • selfishly → *self* (base) + *-ish* (suffix, forming an adjective) + *-ly* (suffix, forming an adverb) • property → *proper* (root) + *-ty* (suffix, forming a noun) • demonstrate → *de-* prefix meaning ‘entirely’ *monstrare-* (root, meaning “to show”) + *-te* (verb suffix) • judgement → *judge* (base) + *-ment* (suffix, forming a noun) • probably → *probable* (base) + *-ly* (suffix, forming an adverb)

The pseudowords were well matched in relation to length (phonemes, letters and syllables) to the real words (see Table 3). This is probably due to the fact that the pseudowords were created by splitting the regular words and recombining them; for example, *yesterday* and *property* gave *yesperty* and *proterday.* This ensured that the pseudowords would preserve some psycholinguistic properties to match the regular/irregular items.

The first and fourth authors created a carefully constructed and 100% agreed-upon bank of phonologically (but not necessarily orthographically) accurate responses, which were used consistently in scoring. Specifically, acceptable spelling responses for nonwords sounded like the target word when read aloud, e.g., ‘clep’ spelled as ‘klep’. Scorers were provided with a list of possible spellings, which are also provided in Appendix E. The pseudowords, apart from phonological knowledge, can also provide insight into the individual’s knowledge of morphemes as, similar to the real words in the list, we have morphologically simple (fourteen items) and morphologically complex words (see Table 5).

**Table 5:** Morphologically simple and complex words in the SPA single-word list (pseudowords)

Pseudowords	
Monomorphemic	Morphologically complex pseudowords
**• lep** **• swem** **• clep** **• prond** **• chust** **• lirst** **• greem** **• shilf** **• grong** **• lorty** **• fize** **• scade** **• scrand** **• bife** **• strofect** **• spantern** **• sombone** **• jubside** **• sandstrate**	• withound → *with* (prefix) + ound (pseudoroot) • prostand → *pro-* (prefix) + *stand* (root) • blarper → blarp (pseudoroot) + *-er* (suffix) • underbably → *under-* (prefix) + bab (pseudoroot) + *-ly* (suffix) • yesperty → yesper (pseudoroot) + *-ty* (suffix) • impabit → *im-* (prefix) + pabit (pseudoroot) • perout → *per-* (prefix) + *out* (root) • trelfishly → trelf (pseudoroot) + *-ish* (suffix) + *-ly* (suffix) • proterday → *pro-* (prefix) + (pseudoroot) • sudgement → sudge *(pseudoroot)* + *-ment* (suffix)

However, as the SPA single-word test was initially developed to assess regular, irregular, and pseudowords, we created an additional strand to our test that specifically targets morphological knowledge, the SPA Morph_test, which we will present next.

### SPA Morph_test development

The SPA Morph_test has 20 real words and 20 pseudowords. Each list has 20 affixes in total and seven suffixation rules, which are scored separately, so the total score an individual can receive is 27 in each list. We do not penalize the speller for errors in the word’s root or pseudoroot, as we are interested in the affixes and the suffixation rules. The morphological spelling test includes three of the four phases proposed for teaching morphological rules by Colenbrander and Kohnen (2021, personal communication). Thus, it has a representation of basic morphological spellings (such as past, continuous tense, plurals, and third-person representation). These items will also be appropriate for Foundation/Key Stage 1 (children who attend the first three years (grades) of primary school). In their second and third phases for teaching morphological rules, the most frequently occurring representations usually taught to Key Stage 2 (children who attend upper primary (elementary) years 3 to 6) children are included, such as prefixes, *re-* (phase 2), *mis-* (phase 3), and suffixes *-ous* (phase 3), and suffixation rules *y to i -ies* (phase 3). We do not include Phase 4 skills as these are more appropriate for Key Stages 3 and 4 children (or upper primary and more into Secondary age). Thus, they will be challenging to master for Foundation/Key Stage 1 children.

Following the same procedure as the SPA single-word test development, a research assistant initially selected items based on the primary school spelling curriculum (DfE, 2013). The research team then reviewed this initial pool, ensuring that two to four items per year group (Reception to Year 6) were matched in terms of frequency, letter count, number of phonemes, syllables, concreteness, imageability and orthographic neighbors (see Table 6 real word comparison across year groups). This ensured that the items remained consistent in key psycholinguistic traits across year groups, and that these traits exerted similar influences on students’ performance across ages. We opted to control for orthographic neighbors that are more appropriate for studying morphological performance because morphological processing is largely based on recognizing written patterns and morphemic structures, whereas phonological neighbors are more relevant to phonologically based spelling skills. The total mean log frequency (based on CPWD, Masterson et al., 2010) was 3.9 *sd* = 1.1. According to the log frequency scale, the items > 2 represent high-frequency words with more than 100 occurrences per million (Masterson et al., 2010). Also, the mean length in letters of the selected words was 7, *sd* = 1.8. This is considered to be a borderline medium to long length according to Brysbaert and New (2009). We carried out the Kruskal-Wallis test, which indicated that selected items were matched across the Year groups in length, frequency, imageability, concreteness and orthographic neighbors, but also that real words and pseudowords were matched in length (letters, syllables and phonemes) and orthographic neighbors (see Table 6, Wilcoxon Z outcome).

**Table 6:** Psycholinguistic characteristic per real words in each year_group and mean pseudowords for the SPA Morph_test

Real words	Log CPWD_a_	N Letters	N Phonemes	N Syllables	Orthog N_b_	Imageability_b_	Concreteness_c_
R Yr (2 items)	4.4 (.21)	6 (.0)	5 (1.4)	1.5 (.7)	7.5 (2.1)	456 (133)	414 (99)
Yr 1 (3 items)	4.4 (1.8)	5.3 (1.1)	4.6 (.57)	1.6 (.57)	4.6 (8.1)	449 (109)	308 (309)
Yr 2 (3 items)	4.1 (.89)	6.6 (1.1)	6 (1)	2.3 (.5)	.66 (1.1)	475 (87)	291 (267)
Yr 3 (3 items)	2.6 (1.2)	8.6 (.57)	6.6 (1.1)	2.6 (.57	.00 (.00)	447 (66)	328 (118)
Yr 4 (4 items)	3.7 (1.5)	6.5 (1.7)	5.2 (.5)	2 (.81)	1.5 (1.9)	461 (47)	291 (302)
Yr 5 (3 items)	4 (.52)	7.6 (3.2)	6.3 (2.1)	2.3 (1.1)	5.6 (8.9)	494 (67)	291 (302)
Yr 6 (2 items)	4.1 (.58)	9 (1.4)	7 (1.4)	3 (1.4)	1 (1.4)	455 (253)	431 (164)
Mean (20 items)	3.9 (1.1)	7 (1.8)	5.8 (1.2)	2.2 (.83)	2.8 (4.8)	463 (89)	276 (228)
Kruskal–Wallis *H real words across year_groups*	5.1	8.01	7.9	5.5	6.3	.79	2.2
*p*	.52	.23	.24	.48	.39	.99	.89
Pseudowords Mean (20 items)	-	7 (1.8)	6 (1.6)	2.2 (.83)	2.8 (4.5)	-	-
Wilcoxon (Z) pseudowords vs. real words	-	1	1.1	.0	.24	-	-
*p*	-	.31	.23	1	.80	-	-

Pseudowords were created by replacing some letters in the root of real words with alternative possible consonants or vowels to create an orthographically plausible pseudoword, but we kept the morpheme unchanged. The decision not to significantly alter the orthographic characteristics of the pseudowords was taken to ensure a close match between the items closely matching real and pseudoword forms (for example, number of letters and phonemes). In this particular task, only the affix and suffixation rule is marked for accuracy, and not the root. For example, *famous* became *pamous*. Both the real word and pseudoword were dictated in a similar way. For example,

- *Winning the final will bring them fame, they will be ****famous. famous****;*
- *Joan uses her pame to sell her stories, she is very ****pamous****. ****pamous***

To ascertain the test’s validity (criterion and convergent), i.e., that the test relates well to other measures that serve the same purpose, we utilized the single-word spelling subtest from the Wechsler Individual Achievement Test – Third Edition (WIAT-3 UK; Wechsler, 2009). This test begins with the assessment of individual graphemes (e.g., <m>). It progresses to single-word spelling with increasingly harder items, and the testing is discontinued after four consecutive errors. The administrator says the target letter or word, followed by a sentence to help disambiguate multisemous items, followed by the repetition of the target item. The highest score a student can receive is 63. The average reliability for 5 to 12-year-olds is α =.94, *sd* =.02.

### Procedure

Ethical approval was obtained from the University’s Ethics Committee before data collection began. Following the school heads’ agreement to testing, parents/carers were asked to consent, and participating children were asked for assent. After ascertaining consent, tests were completed over several days within two weeks in the late Spring and Summer terms (May to June; the last semester of the academic year). The younger year groups completed the spelling tests over several days, with no more than ten items spelled at each time and in small groups of no more than five children. The tests were always administered in groups and in the same order: SPA (irregular, regular and pseudoword items), SPA Morph_test (real and made-up items) and finally, the WIAT3 spelling test. For all tests, the administration followed the same pattern (word- sentence with target word/item – words). Only the SPA pseudowords were repeated three times, without a sentence being given. Learners were asked to listen carefully, but items were repeated as often as needed. Learners were asked to write as clearly as possible, and preferably to avoid cursive writing, for clarity. All tests were administered and scored by paid research assistants who were also primary school teachers (not necessarily working in the participating schools). The research assistants scored all items as correct or incorrect (1/0), apart from the SPA Morph_test (see earlier section), and misspellings were typed into the data sheet. These data were used in the reliability, validity, and standardization analyses presented next.

### Analyses

R (R Core Team, 2026), via RStudio as the integrated development environment (Version 2026.01.0+392; Posit team, 2026) was used for the following analyses (Inferential Statistics, Reliability and Validity).

#### Inferential statistics

To avoid confounding between year group and school or class (each class belonged to a single year group), models did not include school or class random effects. Following sparsity and sensitivity checks, region and SEN status were treated as fixed effects in the inferential models. For SPA and Morph, a random intercept for child accounted for repeated measures across word types. WIAT-3 spelling was analyzed using linear models with year group as a factor, adjusting for region and SEN status.

#### Reliability

Reliability analyses based on Cronbach’s alphas are presented for both tests (SPA single_word and SPA Morph_test).

#### Validity

Criterion and convergent validity were calculated using Pearson’s correlations (*r*) between the spelling tests (SPA single_word, SPA Morph_test, and WIAT3). Age was used as a control variable in the correlation analyses.

#### Standardization

The standardization procedure was automated in a statistical software environment (R; R Core Team, 2026) using RStudio (Version 2026.01.0+392; Posit team, 2026).

Each child’s data underwent a quality check to confirm that the recorded age and raw-score fields contained valid numerical entries. Following this, a stopping rule was implemented for SPA in which any correct answers that followed four consecutive errors were discounted; SPA subscales were then scored according to that process. Then, participants younger than 60 months were removed from the dataset (specifically, 31 participants). Next, a series of Bayesian monotonic models were fitted for SPA subscales and total, and for SPA Morph_test subscales and total. Finally, the simulations were processed to yield raw-score-to-standard-score mappings for 3-month age bins, which were compiled into spreadsheets for easy reference by educators, psychologists, and researchers interested in comparing children’s performance against a developmental trajectory.

#### Differentiation

Multiple case study analyses were used to indicate the differentiation and usefulness of the new tests in comparison to the WIAT3 spelling test.

## Results

### Inferential statistics

#### SPA (binomial mixed model)

There were significant effects of word type, year group and their interaction (word type: χ^2^(2) = 1165.927, *p* <.001; year: χ^2^(6) = 1356.567, *p* <.001; word type × year: χ^2^(12) = 479.327, *p* <.001), with additional fixed effects of region (χ^2^(8) = 78.652, *p* <.001) and SEN status (χ^2^(1) = 28.325, *p* <.001), with lower accuracy in children with SEN relative to those without.

Bonferroni-adjusted pairwise comparisons showed that regular-word accuracy was higher than irregular-word accuracy in every year group (all *p*s <.001). Regular words were also more accurate than pseudowords from year 3 to year 6 (all *p*s <.001), but not in Reception, year 1 or year 2 (*p*s ≥.111). Pseudoword accuracy was higher than irregular-word accuracy in every year group except year 6, where irregular words and pseudowords did not differ (*p* >.999).

**Fig. 2:**
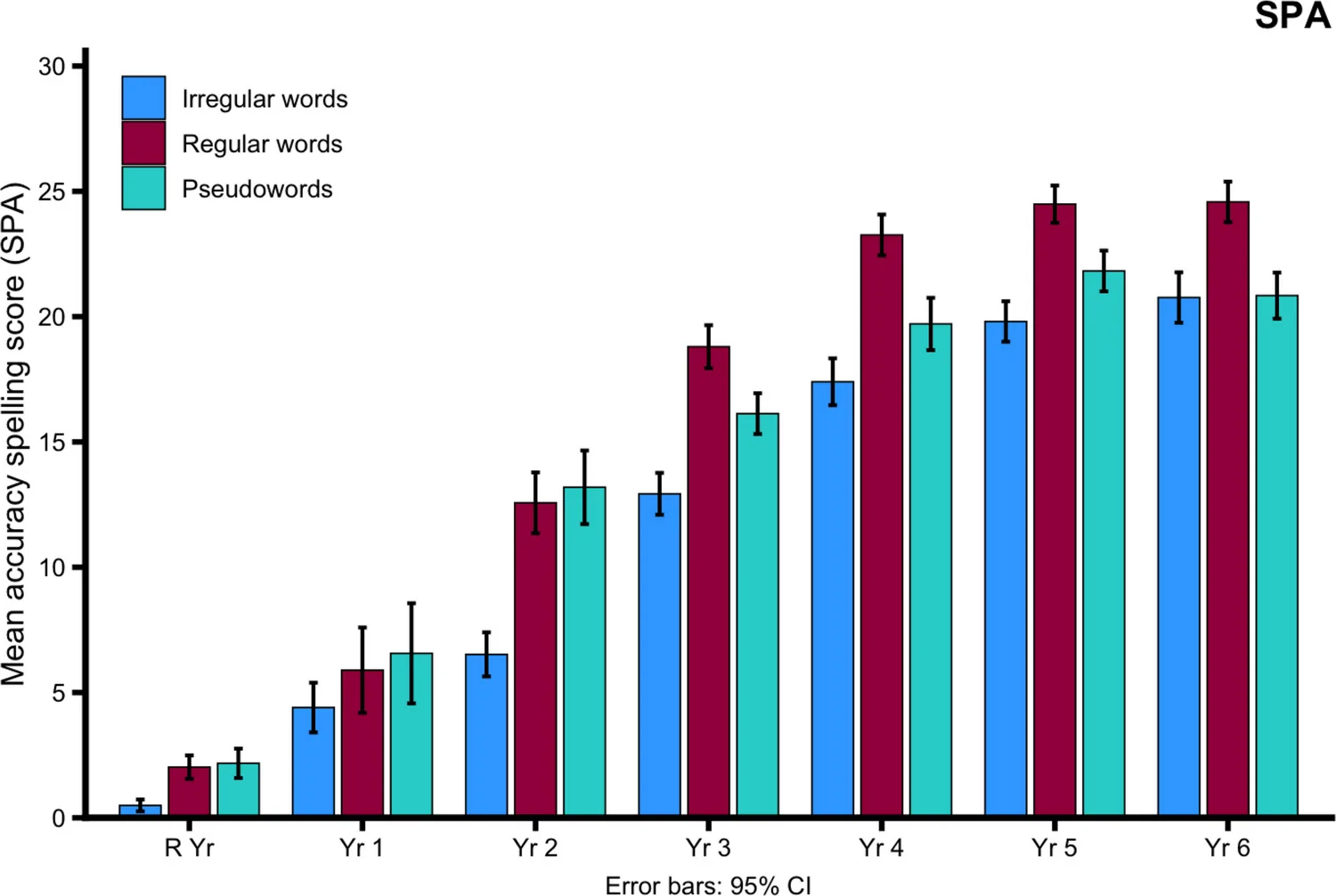
Mean accuracy spelling for regular, irregular and pseudowords (SPA) and interaction with year_group

**Fig. 3:**
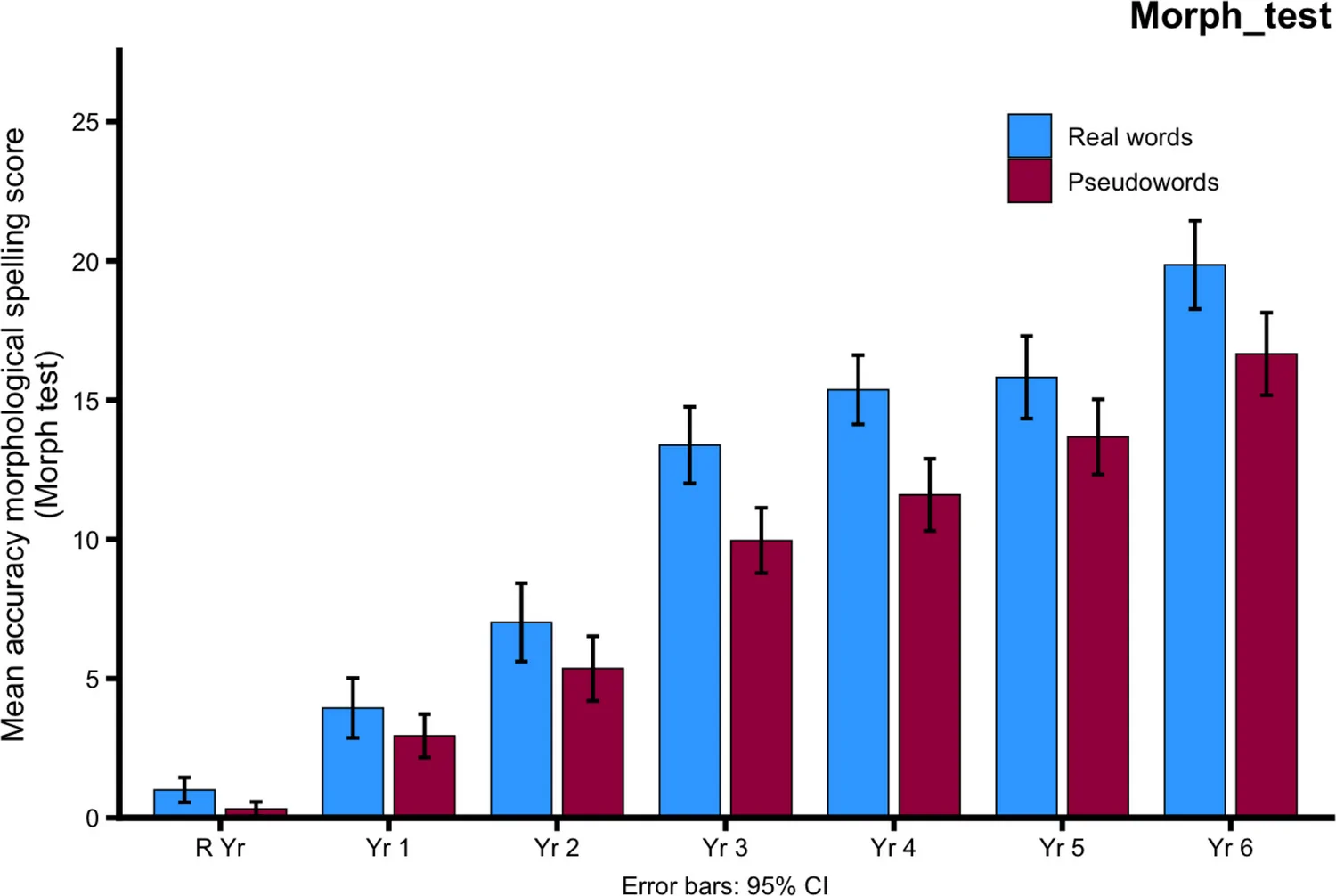
Mean accuracy of morphological spelling for real words and pseudowords and interaction with year_group

**Fig. 4:**
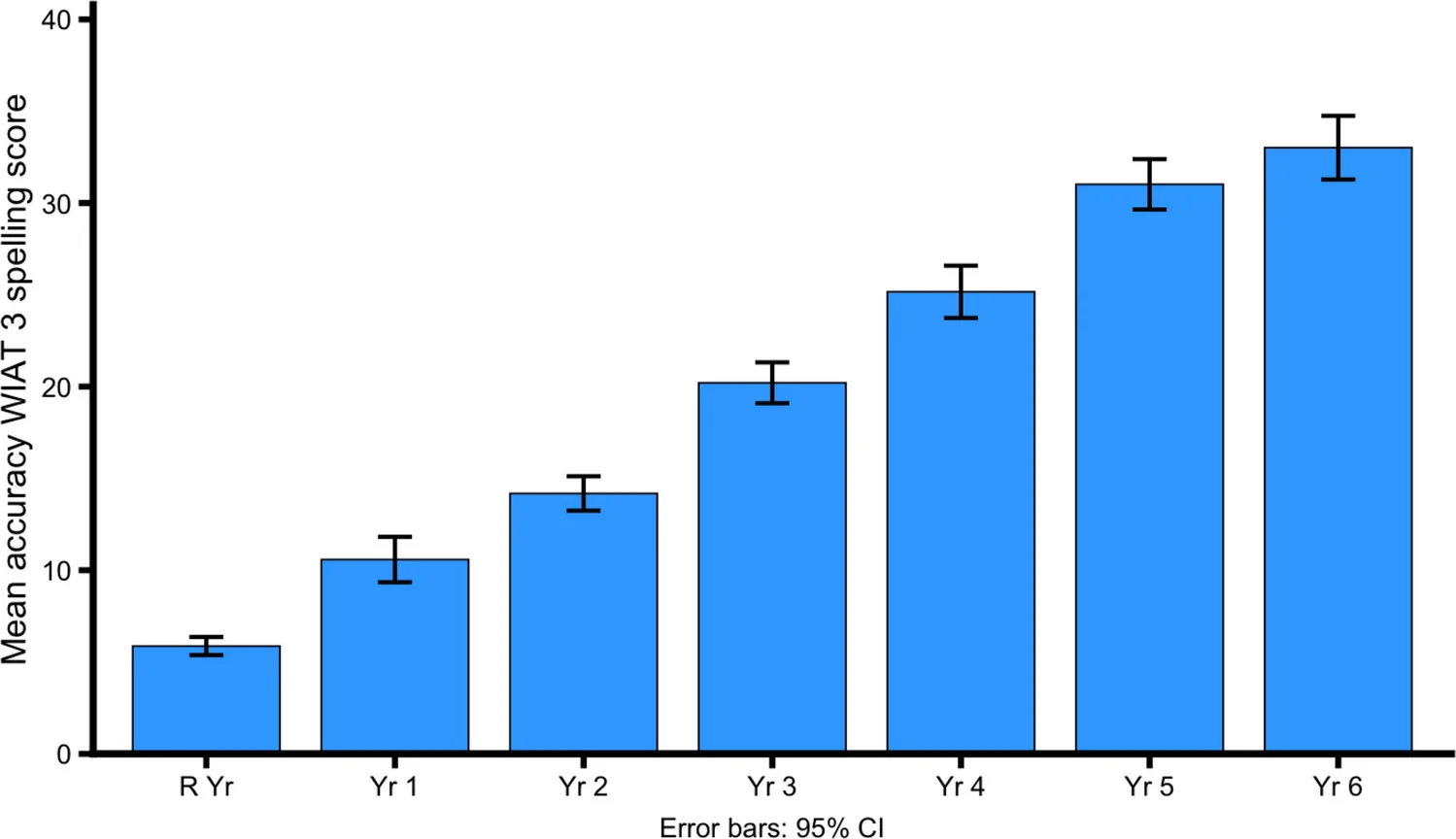
Mean accuracy WIAT 3 single-item spelling subtest and interaction with year_group

Adjacent-year contrasts (Bonferroni-adjusted) indicated improvements across most year boundaries for all word types. The only non-significant adjacent steps were irregular words from year 5 to year 6 (*p* =.094), regular words from year 5 to year 6 (*p* =.850), pseudowords from year 3 to year 4 (*p* =.053), and pseudowords from year 5 to year 6 (*p* >.999) Figs. 2, 3 and 4.

### Morph Test (binomial mixed model)

There were significant effects of morph type, year group and their interaction (morph type: χ^2^(1) = 110.698, *p* <.001; year: χ^2^(6) = 474.152, *p* <.001; morph type × year: χ^2^(6) = 17.355, *p* =.008), with additional fixed effects of region (χ^2^(4) = 55.204, *p* <.001) and SEN status (χ^2^(1) = 23.779, *p* <.001), with lower accuracy in children with SEN relative to those without.

Bonferroni-adjusted pairwise comparisons showed higher accuracy for real-word morphemes than pseudoword morphemes in every year group (all *p*s ≤.013). Adjacent-year contrasts (Bonferroni-adjusted) showed that growth was not significant from year 3 to year 4 and from year 4 to year 5 for either morph type (real word: *p*s >.999; pseudoword: *p*s ≥.492), whereas all other adjacent comparisons were significant (*p*s ≤.035).

#### WIAT-III spelling (raw score; linear model)

There was a significant effect of year group, F(6, 994) = 169.80, *p* <.001, alongside effects of region, F(8, 994) = 7.24, *p* <.001, and SEN status, F(1, 994) = 10.16, *p* =.001, with lower accuracy in children with SEN relative to those without.

Bonferroni-adjusted adjacent-year contrasts indicated that the year 1 vs. Reception difference was not significant (*p* =.115), whereas all later adjacent comparisons were significant (year 2 vs. year 1, *p* =.042; year 3 vs. year 2, *p* <.001; year 4 vs. year 3,* p* <.001; year 5 vs. year 4, *p* <.001; year 6 vs. year 5, *p* =.004).

A linear trend model treating year as a numeric step showed a strong positive trend, *F*(1, 999) = 997.72, *p* <.001, with an estimated increase of 4.83 raw-score points per year step (SE = 0.153, 95% CI [4.53, 5.13]), adjusting for region and SEN status Table 7.

**Table 7:** Mean accuracy (raw scores) of SPAsingle_word, SPA Morph_test and WIAT3 spelling test per year group (standard deviations are in parentheses)

	SPA single_word			SPA Morph_test		WIAT3
Year_group	Irregular words	Regular words	Pseudowords	Real words	Pseudowords	
R Yr	.49 (1.4)	2.02 (2.8)	2.17 (3.4)	1 (1.4)	0.32 (0.7)	5.87 (2.9)
Yr 1	4.4 (3.6)	5.89 (6.3)	6.56 (7.4)	3.94 (3.1)	2.9 (2.2)	10.58 (4.5)
Yr 2	6.52 (5)	12.57 (6.9)	13.19 (8.3)	7.02 (5.6)	5.36 (4.6)	14.18 (5.3)
Yr 3	12.93 (6.5)	18.88 (6.6)	16.13 (6.3)	13.38 (5.8)	9.96 (5.02)	20.21 (8.6)
Yr 4	17.4 (5.8)	23.26(4.8)	19.71 (6.1)	15.37 (5.1)	11.6 (5.3)	25.16 (8.8)
Yr 5	19.8 (5.2)	24.49 (4.8)	21.82 (5.2)	15.82(6.3)	13.68 (5.7)	31.02 (8.8)
Yr 6	20.7 (6.1)	24.58 (4.8)	20.84 (5.5)	19.86 (5.5)	16.66 (5.2)	33.01 (10.4)
Total	12.85 (8.8)	17.34 (9.7)	15.37 (8.9)	11.94 (7.8)	9.46 (6.9)	21.25 (12.04)

### Reliability

Reliability is the consistency of a test’s scores across items and time (Anastasi & Ubrina, 1997). Thus, the test must have appropriate reliability to verify the measure’s consistency. Standard interpretation guidelines are: *α* ≥ 0.9 → Excellent reliability; *α=* 0.8–0.89 → Good reliability; *α=*0.7–0.79 → Acceptable reliability (Kline, 2013). However, Nunnally (1978) suggested that values as low as 0.5 will be acceptable at the early stages of a new test development. We calculated for each item category and each Year group the Cronbach’s *αlpha*_*s,*_ (see Table 8). The lowest score overall, and the only score below.80 is.73 for regular word spelling in year 5, which is still considered acceptable. The total irregular words, regular words, and pseudowords in the SPA test all had excellent reliability: Cronbach’s *α* for irregular words =.99, regular words =.97 and pseudowords =.97.

**Table 8:** Summary of Cronbach’s α results for each item type and each Year_group for the SPA test

	Yr R			Yr 1			Yr 2			Yr 3			Yr 4			Yr 5			Yr 6		
	Ir	Rg	Ps	Ir	Rg	Ps	Ir	Rg	Ps	Ir	Rg	Ps	Ir	Rg	Ps	Ir	Rg	Ps	Ir	Rg	Ps
*α*	.93	.98	.98	.95	.93	.92	.95	.92	.88	.98	.97	.96	.99	.98	.92	.99	.73	.94	.97	.92	.90

Ir = Irregular word; Rg = Regular word; Ps = Pseudoword

Similarly, Cronbach’s *αlpha* reliabilities were calculated for all subtests and for all year groups of the SPA Morph_test (see Table 9). Apart from the pseudoword spelling of the SPA Morph_test in Reception year, which was low *(α* =.46), all other reliabilities were high (*α* >.85). Total real word and pseudoword spelling of the SPA Morph_test all had excellent reliabilities; Cronbach’s *α* for real words =.98 and pseudowords =.97.

**Table 9:** Summary of Cronbach’s α results for each item type and each Year_group for the SPA Morph_test

	Yr R		Yr 1		Yr 2		Yr 3		Yr 4		Yr 5		Yr 6	
	Rw	Ps	Rw	Ps	Rw	Ps	Rw	Ps	Rw	Ps	Rw	Ps	Rw	Ps
*α*	.85	.46	.92	.93	.91	.91	.94	.93	.94	.90	.90	.85	.90	.84

Rw = Real word; Ps = Pseudoword

### Validity

We calculated the test’s criterion and convergent validity as we aimed to identify how well the new test relates to other tests that measure spelling, such as the WIAT3 single-word spelling test. Criterion validity, such as concurrent validity, can verify how well the SPA single_word and SPA Morph_test correlate with a well-established spelling test that was measured simultaneously (Cronbach & Meehl, 1955). Similarly, convergent validity can indicate how well the two new tests (SPA single_word and SPA Morph_test) are theoretically related and how well they correlate with each other (Zeller, 2005). Using data from all children in the standardization sample, in each subtest, we found that all correlations between the SPA test and the WIAT3 test were *r* >.52; these are considered to be high concurrent validity scores (Brysbaert, 2019) (see Table 10). Similarly, the SPA Morph_test correlated highly with the WIAT3 subtest, *r* >.48. While it may be argued that higher correlations are desirable, we would argue that the scores can be interpreted to indicate that the two constructs (SPA single_word and WIAT3 and SPA Morph_test and WIAT3) also tap different aspects of spelling as predicted by our theoretical background. This was, of course, the purpose of the test’s construction. In relation to convergent validity, the association between SPA single_word and the SPA Morph_test is also high, above r>.48. The lowest association was found between SPA irregular word spelling and SPA Morph_test pseudowords, the low correlation is not unexpected, as they tap different constructs in Fig. 1, lexical processing for irregular words and the morphological conversion pathway for morphologically complex pseudowords.

**Table 10:** Criterion and convergent validity Pearson correlations between SPA single_word, SPA Morph_test, and WIAT 3 controlling for the effect of age

	1. Spelling (WIAT3)	2. SPA (Irregular)	3. SPA (Regular)	4. SPA (Pseudoword)	5. SPA total	6. Morph_test real word	7. SPA Morph_test pseudo word	8. SPA Morph_test total
2	0.58****	-						
3	0.53****	0.78****	-					
4	0.53****	0.53****	0.75****	-				
5	0.61****	0.85****	0.95****	0.87****	-			
6	0.50****	0.58****	0.61****	0.58****	0.66****	-		
7	0.58****	0.48****	0.56****	0.60****	0.62****	0.89****	-	
8	0.48****	0.54****	0.58****	0.54****	0.62****	0.79****	0.78****	-

### Standardization

#### Age bins and preliminary filtering

Before modeling, children were provisionally classified into 3-month increments (e.g., 60–62 months, 63–65 months, and so on), ensuring that the final bins reflected tightly defined age intervals. This trimming was performed to avoid age bins in which outliers might disproportionately influence results (Kruschke, 2014). The final standardization dataset (*N* = 985) provided a smooth continuum of ages from youngest to oldest.

#### Bayesian modeling approach

A Bayesian regression model was then fitted to the data to estimate the relationship between children’s raw scores and their age bins. The modeling framework allowed the location (mean) of the distribution and its scale (variability) to change in a monotonic fashion with increasing age (Bürkner, 2017, 2018). For example, this meant that older bins were expected to have scores that were equal to or higher than younger bins, unless the data strongly suggested otherwise. By using a partially pooled model, each bin’s parameter estimates were informed not only by the children in that bin, but also by data from adjacent bins (Gelman & Hill, 2007). SEN status was not included in the norm-generating model, because the published standard-score tables are intended as general age-based reference norms rather than SEN-adjusted norms. The standard-score tables are age-based reference norms for the standardization sample, intended to support identification of children whose spelling performance is low relative to age expectations.

An additional advantage of this framework was the inclusion of a school-level random intercept to account for the clustered nature of the calibration sample, helping to ensure that the overall developmental trend was not skewed by a small number of schools with unusually high or low scores. This arrangement provided an extra layer of stability to the estimates, especially in instances where certain schools had fewer participants than others. The published lookup tables were generated using population-level predictions, with school-specific random effects omitted; therefore, the resulting norms are not conditional on any particular school. Furthermore, we allowed the amount of unexplained variation to change steadily with age, because older pupils might not only score differently on average, but their scores might be more (or less) spread out.

#### Posterior predictions and standard score conversion

After the Bayesian model converged on stable parameter estimates (4000 ‘warm-up’ iterations), 4000 simulations were drawn from its posterior predictive distribution, using a Hamiltonian Monte Carlo sampler (Stan Development Team, 2022). These simulations represented plausible raw-score outcomes for each age bin in the dataset, given the fitted age trajectory and the partial pooling constraints. To create a lookup table, each possible raw score was matched to the simulated distribution. The proportion of simulations at or below each raw score was taken as a cumulative probability. Next, these probabilities were transformed to *z*-scores and then linearly scaled so that the resulting standard scores had a mean of 100 and a standard deviation of 15. Most tests use standard scores to show the individual’s position compared to peers of the same age. These are considered the most appropriate for providing a picture of individuals who perform lower or higher by 1, 2, or 3 *Sds* than the mean on the normal distribution curve (Patoss, 2025).

This conversion step is analogous to classical *z*-scoring, but rather than calculating separate means and standard deviations per bin in isolation, the Bayesian model enforced consistency across age bins (Carlin & Louis, 2008): for example, a raw score of 50 at age 9 will always result in a standard score close to what would be expected for that age, even if adjacent bins have small differences in raw score distributions. The Bayesian approach models the relationship between raw scores and age (or other predictors) as part of a continuous developmental trajectory: rather than treating each bin independently, the model uses partial pooling to share information across bins while still allowing for differences between them. This is achieved by modeling age as a coherent developmental trajectory within a single Bayesian model, which shares information across adjacent bins while still allowing age-related differences in the implied raw-score distribution. Instead of relying solely on observed raw scores to compute μ and σ within each bin, the Bayesian model generates thousands of posterior predictive samples for each bin. These samples represent the uncertainty in the data and ensure that the final standard scores reflect the variability and uncertainty in the observed scores.

The final steps involved clamping extreme values (e.g., those below 55 or above 145) to ensure the lookup tables did not present implausibly extreme values and then applying a smoothing algorithm to force row-wise monotonicity such that the standardized score for any raw score always increased or stayed the same with increasing age. We reshaped the standard‐score table into a simple number matrix and then ran an isotonic regression (the pool‐adjacent‐violators algorithm) to smooth out any dips so that scores never fall as age increases. After smoothing, we converted the numbers back into our table layout and rounded everything to whole points, resulting in a clean, non‐decreasing progression of standard scores across all age intervals. The end-product was a table of raw-score entries that, for each 3-month interval, indicated an expected standard score (open science link). Similarly, year/grade-based norming tables have been calculated and added to our online open science materials.

#### Differentiation (case series analyses year 3)

In this final part of the results section, we present scores from the WIAT3 alongside results from the SPA single word (pseudowords, regular words, irregular words) and the SPA Morph_test (words and pseudowords) administered to four individuals. At the time of the assessment, the children attended Y3, and their ages ranged from 8.3 to 8.5 years. See also Table 11. We will treat scores that are 1SD lower than the mean (SS below 85) as indicating a need for intensified support, while also acknowledging the fact that these cut-offs are arbitrary and scores of (and even above) 85 may still require some support.

**Table 11:** Results of case series analyses for all spelling sub-tests/tests and composite scores

	SPA single_word test SS				SPA Morph_test			WIAT-3
Participant	Irregular	Regular	Pseudowords	SPA Composite score	Real words	Pseudo words	SPA Morph_test Composite score	
Male A Y3 (8.4 years)	82	85	84	83	78	86	82	77
Female A Y3 (8.3 years)	74	78	95	82	85	89	87	80
Female B Y3 (8.5 years)	82	85	106	91	87	95	89	94
Male B Y3 (8.3 years)	85	107	106	98	102	109	103	98

We first present Male A, who has a diagnosis of ADHD, but not dyslexia (or spelling impairment), and he presents with a below-average spelling score on the WIAT3. While this score indicates a difficulty with spelling, it’s unclear what exact difficulties Male A has. The SPA indicates challenges across the items tested, with slightly more difficulties in real-word than pseudoword spelling. For him, an intervention targeting sublexical (phoneme-grapheme correspondences and orthographic patterns) and lexical/semantic processes and morphological spelling knowledge will likely be very helpful.

Female A did not have a diagnosis of literacy difficulties, but, similar to Male A, she scored below average on the WIAT3. Again, while this indicates a difficulty with spelling, it does not tell us where these difficulties lie. It could be that there are spelling difficulties across the board, as seen in Male A. However, in contrast to Male A, the SPA actually indicates that she struggled significantly with irregular and regular word spelling and with spelling of morphological patterns when this involved real words. Her pseudoword spelling accuracy was within the average range on the SPA, as was her spelling of morphologically complex pseudowords. Her overall strength in pseudowords could potentially indicate that phonics interventions put in place for her worked well, but this strength did not help her when spelling real words (regular [e.g., *blade* as *blayd*, *prize* as *prise*] and irregular [e.g., *said* as *sed*, *deadly* as *dedly*). Real words, in contrast to pseudowords, also tap orthographic patterns and lexical/semantic processes, so a pure phonological approach would not be enough to accurately spell words that span a continuum of transparency or regularity. This shows in her responses, which are phonologically plausible yet inaccurate spellings.

Female B does not have any diagnosed literacy difficulties, but she is on the free school meal (FSM) register. Although Female B presented with a spelling profile on that SPA that is similar to that of Female A, her WIAT3 spelling score did not fall below the cut-off for single-word spelling. The SPA test results indicate that for these two individuals (Female A and B), phonics intervention at this point in their spelling acquisition might not be enough to make a significant change. Mainly, errors in Female B’s responses were phonologically appropriate already. Explicit work on spelling patterns and rules, including morphological spelling patterns (e.g., *kicked* as *kict*; *harmless* as *harmles*) and word-specific orthography to spell irregular words (some irregular word spelling errors were *analyze > analiys* and *councilor > canseler*), would target current gaps in Female B’s spelling knowledge.

Finally, Male B does not have a SEN diagnosis, but he is on the FSM register. His WIAT3 scores are in the average range, suggesting that there is no spelling difficulty. However, on the SPA, he presents with some challenges in irregular word spelling only, so an intervention targeting spelling of commonly occurring irregular words (lexical-semantic route) will likely be helpful. His errors were mainly phonologically appropriate ones (*fortune > forchun*). Explicit teaching of irregular words that are high in frequency or relevant to his schoolwork is likely most appropriate. This could be done via repeated exposure and copying (Brunsdon et al., 2005), spelling patterns using visual imagery, e.g., the word ‘look’ having two eyes in the middle to remember the ‘oo’ (Niolaki et al., 2021; Schmalzl & Nickels, 2006), dot-and-check spelling (Weakland, 2021), or spelling pronunciations (Lithgow et al., 2024; 2026) may be helpful. Note that the WIAT3 missed Male B’s difficulty with irregular word spelling. We think the case studies illustrate well the utility of the SPA for precise identification of spelling difficulties and its role in directions for intervention.

## Discussion

The paper presented the development and standardization of a new single-word spelling battery that includes, in part a., irregular, regular, and pseudowords (SPA single word) and part b. a morphological spelling test spanning both real words and pseudowords (SPA Morph_test). The test construction is theoretically driven, largely based on Dual Route theory with amendments made to integrate morphological processing, including findings by researchers within the integration of multiple patterns theory. Items were carefully selected to tap different spelling skills (phonology, orthography, semantics, and morphology) to align with current theories of spelling. The test aims to provide a theoretically based measurement of spelling skills to better assist in formulating intervention targets for children who struggle with spelling.

The test was normed on a large number of children in the UK, allowing us to provide normative comparison data and explore the reliability and validity of the SPA. The SPA single-word and the SPA Morph_test, exhibit good reliability and validity. The SPA Morph-test pseudowords have low reliability in the Reception Year children, but this is not unexpected as this is a new tool (Nunnally, 1978). Further, children as young as 5 years often do not receive explicit instruction on morphological rules, and hence their proficiency will be lower than other age groups and likely more variable, too. Despite their low reliability in the early years, the strength of using pseudowords lies in their ability to measure processing without also tapping into lexical knowledge. This makes pseudowords valuable not only for research purposes but also for clinical and school-based testing, as they allow us to evaluate if patterns and rules can be generalized to untaught or unfamiliar words. More generally, the SPA provides a set of spelling tests assessing a wide range of linguistic elements within a single tool. Typically, educators need to purchase several different assessments to achieve the same comprehensive evaluation that our tool provides (Kohnen et al., 2009). In addition, although the test demonstrates strong criterion and convergent validity, the results also show that the different item lists distinctly tap into separate linguistic processes: phonology, orthography, and morphology. This is evident in the lack of perfect correlations among these different subtests, with the weakest association observed between irregular word reading and pseudoword decoding in our analyses.

We confirmed that children’s spelling on the SPA improves gradually over the primary school years. Some word types are more challenging to spell than others (pseudowords and regular words initially are easier, irregular words are harder). Children’s irregular word spelling is on a par with the other item types only at the end of primary school. This could be due to many factors, one of which may be the UK’s phonics-heavy curriculum, as well as the lack of a spelling policy in most UK schools and the way instruction is primarily focused on rote learning of weekly lists that have low generalizability in spelling and writing, and they are practiced only weekly and at home (see reviews by Cawley et al. (2026) and Esposito et al. (2023). The lack of long-term spaced repetition and recall practice of the weekly spelling list, using different methods (flashcards, concept cards, spelling and writing, among others), can negatively affect retention in long-term memory, as shown by Ebbinghaus (2013) in the *forgetting curve*.

The other goal for this test was to produce normative comparison data for practical use in schools and clinics. To convert diverse raw scores into a single age-referenced scale, we adopted a Bayesian-hierarchical scoring pipeline that replaced the usual “one-bin, one-z” routine with a smooth developmental curve that pooled evidence across age bands, schools, regions, years and SEN status, thereby taming the random bumps that arise when some subsamples are thin (Gelman & Hill, 2007). By constraining both the mean and the spread of scores to change gradually with age and by projecting thousands of posterior simulations onto the familiar 100 ± 15 metric, the method produced norms that were far steadier than the jagged, sometimes backward-stepping values of classical tables. A final light-touch smoothing algorithm subsequently enforced strict monotonicity, ironing out the rare instances where very sparse cells still produced reversals. Every step, from raw data to lookup sheet, was scripted in freely available code, so the entire pipeline remains transparent, reproducible, and easy to adapt.

A brief case series demonstrates the applicability of the tool. The SPA Single_word/Morph_test was able to identify difficulties with more precision than the WIAT3. The case series results show that a commonly used single spelling tool such as the WIAT3 can miss struggling spellers altogether or identify them as struggling without pinpointing the skill deficit these students have. Without knowing which aspects of spelling are affected, it is impossible to target intervention effectively. The SPA battery also provides rich qualitative insights, supported by a detailed spelling guide (forthcoming in a companion monograph) that helps educators identify the specific processes and rules a learner may not yet have fully mastered. This approach aligns beautifully with current approaches that shift the focus from penalizing mistakes to empowering learners with the tools and knowledge they need to master spelling with confidence.

As with any newly developed tool, certain limitations need to be acknowledged. Some lower reliability scores are to be expected (one in year 5 regular words (SPA single-word test) and another one in Reception Year Pseudoword (SPA morph-test), given the novelty of the tool and variations in instruction. Additionally, the assessment focuses on accuracy rather than fluency. However, fluency is often considered a goal that needs to follow accuracy, and can still be observed qualitatively through the learner’s behavior, such as the speed of their spelling retrieval or the frequency with which they cross out and correct their responses.

It is also important to acknowledge that the measures of concreteness and imageability were taken from adult databases rather than child databases, as no such data have been reported to date in child databases. However, because both psycholinguistic variables are important for memory processes and learning, concrete words (e.g., *bag*) and highly imageable words (e.g., *swim*) are more likely to be encoded correctly (Brysbaert et al., 2014). Even with the caveat that we relied on adult databases, no significant differences were detected.

The pseudowords were created by either transposing parts of the regular words (from the SPA single-word test) or by changing some letters in the root of the pseudoword (SPA Morph_test). This could potentially mean that the items can be spelled by analogy to the real words. However, for the SPA Morph_test, only the morphological part is scored for correctness, and we wanted the child’s focus to be on the morphological part of the word rather than the correct spelling of the root, meaning that we could effectively match the items while also minimizing orthographic memory demands.

## Concluding considerations

By adopting a Bayesian monotonic approach, the resulting standard score tables preserve a smooth pattern of growth in average performance and variability across ages. This method offers a potential improvement over strictly binned *z*-score norms, as it reduces abrupt shifts from one bin to the next and integrates partial pooling to mitigate instability in smaller groups (Gelman & Hill, 2007). The resulting norms are intended to provide a more coherent picture of children’s spelling development, helping practitioners identify relative strengths and weaknesses when interpreting individual assessment outcomes. This is an essential step in providing targeted intervention. Overall, we have developed a theoretically grounded assessment battery that captures the many linguistic elements learners draw upon when spelling. With this tool, we aim to empower teachers, assessors, and researchers to give spelling, the often overlooked component of literacy, the attention it deserves.

## Supplementary Information

Below is the link to the electronic supplementary material.

Supplementary file1 (DOCX 738 KB)

## Data Availability

Synthetic data that capture the key characteristics of the original dataset and materials are available at https://osf.io/vfp7h/files/osfstorage.

## Footnotes

1
*That another relevant question regarding the exact nature of the morphological route is how meaning-carrying affixes are linked to semantics.*

## Code availability

Documented code for the analyses in this article is available at (https://osf.io/vfp7h/files/osfstorage).
