# Supplementary material for: Spelling Profile Assessment (SPA): A new spelling test for English primary school children; standardization
Source: Behav Res Methods. 2026 Nov 9;58(12):348. doi: 10.3758/s13428-026-03195-7 (PMC13748090; doi:10.3758/s13428-026-03195-7)
Supplement: Supplementary file 1 [file 13428_2026_3195_MOESM1_ESM.docx]

**Appendix**

**Appendix A**

*The Item Characteristic Curves (ICC) per type of word (irregular (a), regular (b) and pseudoword items (c)*

a. *Irregular*

b. *Regular items*

c. *Pseudowords*

**Appendix B**

*Reliability based on the* ***pilot study***

|  | ***Irregular 36*** | ***Regular 36*** | ***Pseudowords 34*** |
| --- | --- | --- | --- |
| *TOTAL*  *N=641* | *0.97* | *0.96* | *0.94* |
| *YEAR RYr* | *0.72* | *0.85* | *0.82* |
| *YEAR 1* | *0.92* | *0.9* | *0.89* |
| *YEAR 2* | *0.94* | *0.92* | *0.88* |
| *YEAR 3* | *0.92* | *0.89* | *0.9* |
| *YEAR 4* | *0.93* | *0.9* | *0.79* |
| *YEAR 5* | *0.94* | *0.85* | *0.72* |
| *YEAR 6* | *0.87* | *0.78* | *0.71* |

**Appendix C**

*Criterion and Convergent validity* ***Pilot study****, controlled for age (total items SPA 106)*

|  | *1. total spelling SPA* | *2. spelling (Irregular)* | *3. Spelling (Regular)* | *4. Spelling (Pseudowords )* | *5. Spelling (WIAT 2)* |
| --- | --- | --- | --- | --- | --- |
| *1* | *-* |  |  |  |  |
| *2* | *.87**** | *-* |  |  |  |
| *3* | *.94**** | *.76**** | *-* |  |  |
| *4* | *.85**** | *.53**** | *.73**** | *-* |  |
| *5* | ***.78****** | ***.77****** | ***.74****** | ***.57****** | *-* |

**Appendix D**

*Psycholinguistic characteristic per item type (irregular, regular and pseudowords)* ***Pilot phase***

|  | *CPWD_a_* | *CBeebies_b_* | *CBBC_b_* | *Zipfreq_b_* | *N Letters* | *N Phonemes* | *NSyllables* | *CD CBBC_b_* | *CD zip_b_* | *Phon N_c_* |
| --- | --- | --- | --- | --- | --- | --- | --- | --- | --- | --- |
| *Irregular items* | *416 (880)* | *4.15 (.97)* | *4.26(.92)* | *4.32 (.96)* | *6.25 (2.0)* | *5.47 (1.75)* | *1.75 (.81)* | *.14(.19)* | *.17(.21)* | *6.4 (7.3)* |
| *Regular items* | *128 (214)* | *4.08 (1.3)* | *4.42(1.2)* | *4.52 (1.1)* | *6.8 (1.7)* | *5.3 (1.7)* | *2.1 (.68)* | *.2 (.25)* | *.26(.28)* | *3.9 (4.3)* |
| *pseudoword* | *-* | *-* | *-* | *-* | *6.4 (2.0)* | *5.6 (2.0)* | *1.8 (.9)* |  |  |  |
| *Kruskal-*  *Wallis H* | *.09* | *.002* | *1.06* | *.65* | *2.05* | *1.13* | *5.09* | *.96* | *1.37* | *.46* |
| *p* | *.76* | *.96* | *.30* | *.42* | *.35* | *.56* | *.08* | *.32* | *.24* | *.49* |

*Note: a= Masterson et al. (2010); b=Van Heuven et al. (2014); c=Davis (2005)*

**Appendix E**

***Scoring of Nonwords***

**Scoring:** The suggested alternative spellings and presentations are deemed as acceptable: (an acceptable non-word sounds like the non-word pronunciation when read aloud but it is spelled differently (like clep spelled as klep). Note that this means the acceptable spelling may have sequences of letters that you would not often see in real words (e.g., chusst, shillf, withownd, sckrand, scayd). However, if the same sound is represented twice (i.e. there is no accepted doubling grapheme), such as the w in swwem, or by a mix of two graphemes e.g., the /or/ in lawghty presumably a combination of aw and ough, this is incorrect, or by an incomplete grapheme e.g., /or/ in laugty as aug, presumably missing h). **Please note that the below is not a complete list of plausible spellings, but a set of examples.**

lep: /l/ [l] - /ep/ [ep, epp]

swem: /s/ [s] - /w/ [w, wh] - /em/ [em, emm, emb]

clep: /k/ [c, k] - /l/ [l] - /ep/ [ep, epp]

prond: /pr/ [pr] - /ond/ [ond, onned, onnd]

chust: /ch/ [ch] - /ust/ [ust, ussed,]

lirst: /l/ [l] - /ir/ [ir, er, ur, irr, err, urr, ear] - /st/ [st, sed]

greem: /gr/ [gr] - /eem/ [eem, eam, eme, eeme, eame]

shilf: /shi/ [shi] - /l/ [l, ll] - /f/ [f, ff, ph]

grong: /gr/ [gr - /ong/ [ong]

lorty: /l/ [l] - /or/ [or au augh aw ough oor our] - /ty/ [ty tey tee tea ti tie]

fize: /f/ [f, ph] - /ize/ [ize, ise, iyz, iys, yze, yse, ighs, ighz, iese, ieze, ies, iez, eyes, eyez]

scade: /s/ [s] – /k/ [c, k, ck] - /ade/ [ade, ayd, ayed, aid, aied, aide]

scrand: /s/ [s] - /c/ [c, k, ck] - /rand/ [rand, anned]

bife: /b/ [b] - /ife/ [ife, yf, yfe, ief, ieff, ighf]

withound: /w/ [w, wh] - /ith/ [ith] - /ou/ [ou, ow] - /nd/ [nd, ned, nned]

prostand: /pro/ [pro] - /st/ [st] - /and/ [and, anned]

strofect: /stro/ [stro, strough] - /f/ [f, ff, ph] - /e/ [e] - /ct/ [ct, ckt, kt, cced, cked]

blarper: /bl/ [bl] - /ar/ [ar, arr, ah] - /p/ [p, pp] - /er/ [er, ur, a, or]

spantern: /span/ [span, spann] - /ter/ [ter, tir, tur, tir] /n/ [n, nn]

underbably: /und/ [und] - /er/ [er, ur, ir, e, u, i, ea] - /bab/ [bab, babb] - /ly/ [ly, ley, lee, lea, lie]

sombone: /som/ [som, somm] - /bone/ [one, own, oan,]

jubside: /jub/ [jub] - /side/ [side, cide, sied, cied, syed, sighed]

yesperty: /ye/ [ye] - /s/ [s, ss] - /per/ [per, pur] - /t/ [t, tt] - /y/ [y, ee, ea i, ey, ie]

sandstrate: /sand/ [sand] - /str/ [str] - /ate/ [ate, ait, ayt, eyt, aight, eight, aite]

impabit: /imp/ [imp] - /a/ [a] - /bit/ [bit, bet, bitt, bett, bbit, bbet ]

perout: /p/ [p] - /er/ [er, ar, ir, ur] - /out/ [out, owt]

trelfishly: /tr/ [tr] - /e/ [e, ea] - /l/ [l. ll] - /f/ [f, ph] - /ish/ [ish] - /ly/ [ly, ley, lee, lea, lie]

proterday: /pro/ [pro] - /t/ [t, tt] - /er/ [er, ur, ir, urr, e, u, i, ea] - /day/ [day, eigh, aigh, ey, ai]

sudgement: /su/ [su] - /dge/ [dge, dg, dj, dje, je, j] - /ment/ [ment, munt, mant, meant]
